# Effects of Pretreatment Methods on Gamma-Aminobutyric Acid Enrichment and Quality Improvement in Highland Barley Beverages

**DOI:** 10.3390/foods13244053

**Published:** 2024-12-16

**Authors:** Xiaoqing Yin, Shanshan Wang, Zhirong Wang, Huaying Wen, Ting Bai, Yuhong Zhang

**Affiliations:** 1 Institute of Food Processing, Xizang Academy of Agricultural and Animal Husbandry Sciences, Lhasa 850000, China; keyanren_xiaoqing@163.com (X.Y.);; 2 School of Food Science and Engineering, Yangzhou University, Yangzhou 225127, China

**Keywords:** highland barley, enzymatic hydrolysis, GABA, antioxidant activity, flavor, beverages

## Abstract

Gamma-aminobutyric acid (GABA) is an important neurotransmitter that promotes sleep and reduces anxiety, but its natural synthesis in the body is insufficient, necessitating dietary intake. This study utilized a combination of germination, the addition of active barley powder, and fermentation to enhance GABA content in an enzymatically hydrolyzed highland barley beverage. The samples were divided into five groups: highland barley (HB), germinated highland barley (GB), highland barley supplemented with another high-glutamic-acid decarboxylase-active highland barley powder TB13 (BT), germinated barley supplemented with TB13 (GBT), and germinated barley supplemented with TB13 followed by fermentation (GBTF). The results indicated that all the pretreatments significantly elevated GABA levels, with the GBT sample showing the highest GABA content, which was 2.4 times that of the HB sample. Germination had minimal impact on the taste and aroma of the beverage, while the addition of TB13 active barley powder caused only slight changes to the aroma. The GABA content in the GBTF sample was 2.2 times higher than in the HB sample, and the GBTF sample also exhibited the highest total phenolic content, demonstrating the strongest antioxidant and free-radical scavenging abilities. Furthermore, the GBTF treatment increased acidity, reduced bitterness, and significantly altered the flavor profile of the barley beverage, enhancing its overall quality and consumer appeal as a GABA-rich functional drink.

## 1. Introduction

Highland barley, an ancient cereal grain, has been a staple in human diets for millennia, renowned for its rich nutritional profile and health benefits [1,2]. In recent years, there has been growing interest in the development of functional beverages derived from barley, particularly those enriched with bioactive compounds, such as γ-aminobutyric acid (GABA). GABA, a nonprotein amino acid, is known for its role in promoting relaxation, reducing stress, and improving sleep quality, making it a highly desirable component in health-oriented products [3,4]. Due to extreme climatic conditions, such as intense UV radiation, drought, and hypoxia [5], the GABA content in highland barley ranges from 18 to 28 mg/100 g, which is higher than that in rice (10.75 to 18.62 mg/100 g) [6], soybeans (2.99 mg/100 g) [7], and wheat (0.12 mg/100 g) [8]. However, it is still necessary to employ various pretreatment methods to increase the concentration of GABA in highland barley.

Different pretreatment techniques, including germination, fermentation, and enzyme hydrolysis, have been explored for their potential to increase GABA levels in barley. The main metabolic pathway of GABA in plants and lactic acid bacteria is the GABA shunt, and the polyamine degradation pathway is also one of the synthesis pathways of GABA in plants [9,10]. During germination, enzymes such as proteases and glutamic acid decarboxylase (GAD) in highland barley are activated, leading to protein hydrolysis and an increase in glutamate content, which is further converted into GABA under the action of GAD [11]. Sprouting not only increases the GABA content in HB but also enhances the antioxidant activity of free phenolic compounds and their end products [12]. Phenolic compounds in barley itself can directly participate in scavenging free radicals, transforming peroxyl radicals into stable byproducts, or indirectly clearing free radicals by inhibiting enzymes that can generate free radicals [13]. Interestingly, different varieties of highland barley exhibit varying levels of GAD activity, with higher GAD activity favoring GABA enrichment [11]. Therefore, adding barley flour with a high GAD enzyme activity is also a new strategy for enhancing GABA levels. Fermentation further boosts the nutrition, digestibility, and sensory properties of cereal beverages, facilitating easier absorption of antioxidants and micronutrients while reducing the content of antinutrients (such as phytic acid) and substances like hexanal, which may lead to undesirable flavors [14].

The application of these pretreatment methods has implications not only for GABA enrichment but also for the overall quality of the barley beverage. Factors such as taste, aroma, texture, and shelf life are critical to consumer acceptance and can be significantly influenced by the chosen pretreatment strategy. For instance, fermentation can enhance flavor profiles by introducing desirable organic acids, while germination can improve mouthfeel by increasing the soluble fiber content. Therefore, optimizing these pretreatment processes is essential to developing a barley beverage that is both nutritionally enhanced and organoleptically appealing.

This study aims to investigate the effects of various pretreatment methods on the enrichment of GABA in barley beverages, with the goal of identifying the most effective strategies for producing a functional barley beverage that meets the increasing consumer demand for health-promoting products.

## 2. Materials and Sample Preparation

### 2.1. Materials and Chemicals

The experimental material, Tibetan highland barley variety (Zangqing 2000), was cultivated in Lhasa, Tibet, in 2017 at an altitude of 3658 m. The oxygen content at this high altitude was 15.09%, with an average temperature of 18.6 °C. The whole barley powder, obtained through cyclone grinding, was stored in a self-sealing bag in a refrigerator at 4 °C.

GABA, 4-dimethylaminobenzene-4′-sulfonyl chloride, acetonitrile, and methanol were purchased from Aladdin (Shanghai, China). The amino acid assay kit, flavonoid assay kit, and DPPH radical scavenging capacity assay kit were obtained from Solarbio (Beijing, China). The total antioxidant assay kit, hydroxyl radical scavenging capacity assay kit, superoxide anion radical scavenging capacity assay kit, and total phenol assay kit were purchased from Nanjing Jiancheng Bioengineering Institute (Nanjing, China). Cellulase was provided by Heshibi Biotechnology Co., Ltd. (Beijing, China). High-temperature α-amylase, flavor protease, and amylase were supplied by Novonesis (Beijing, China) Biotechnology Co., Ltd. All other chemicals and reagents used in the experiments were of analytical grade and were purchased from Kelon Chemical Reagents Factory (Chengdu, China).

### 2.2. The Germination Process of HB

The processed barley was soaked for 6 h and germinated in a humidity chamber with constant temperature and humidity at 30 °C for 36 h, and then the temperature was adjusted to 50 °C for an additional 2 h of germination. Afterward, the samples were sun-dried for approximately 3 to 5 days until the moisture content in the highland barley powder reached 7–9%.

### 2.3. Sample Coding and Pretreatment Preparation

#### 2.3.1. HB Powder

Fifty grams of highland barley powder was added to water in a 1:10 ratio and pregelatinized at 85 °C for 10 min. Alpha-amylase was added at a dose of 0.5‰ and enzymatically hydrolyzed at 85 °C for 2 h (enzymatic hydrolysis phase 1). Then, this was cooled to 50 °C, glucoamylase was added at a dosage of 1.8‰, 0.18 g/mL of flavor protease was added at a dosage of 6‰, and 0.045 g/mL of cellulase was added at a dosage of 1‰. Enzymatic hydrolysis was continued for 2 h (enzymatic hydrolysis phase 2). This solution was then centrifuged at 4000 rpm for 5 min, and the supernatant was taken, sealed, and sterilized, and its enzymes were deactivated in a 90 °C water bath for 30 min and cooled before being stored in the refrigerator. Untreated highland barley samples were evaluated as controls and coded by HB.

#### 2.3.2. Germinated Highland Barley Powder

Highland barley powder was replaced with germinated highland barley powder (50 g), and the same procedures as described in Section 2.3.1 were followed. This sample group was coded as GB.

#### 2.3.3. Highland Barley Powder: TB13 Addition

After 1 h into the second stage of enzymatic hydrolysis, 5 g of active highland barley powder (TB13) was added and mixed thoroughly, and enzymatic hydrolysis was continued. The same procedures as described in Section 2.3.1 were followed. Samples for this pretreatment were coded as BT.

#### 2.3.4. Germinated Highland Barley Powder: TB13 Addition

We substituted 50 g of germinated highland barley powder for highland barley powder, and, after 1 h into the second stage of enzymatic hydrolysis, we added 5 g of TB13, mixed thoroughly, and continued enzymatic hydrolysis. We followed the same procedures as described in Section 2.3.1. This sample group was coded as GBT.

#### 2.3.5. TB13 Was Added and Fermented After Enzymatic Digestion

We substituted 50 g of germinated highland barley powder for highland barley powder, and, after 1 h into the second stage of enzymatic hydrolysis, we added 5 g of TB13, mixed thoroughly, and continued enzymatic hydrolysis. After completion of enzymatic hydrolysis, we conducted high-pressure sterilization. After cooling, we inoculated Lactobacillus plantarum (1‰ g/mL) with 20 uL in a sterile laminar flow hood. We added Tianjiuqu at a rate of 4‰ (0.2 g) and cultivated it in a constant-temperature incubator at 36 °C for 24 h. After high-pressure sterilization, it was centrifuged at 4000 r/min for 5 min, the supernatant was collected and sealed, and we performed sterilization in a water bath at 90 °C for 30 min, left it to cool, and stored it in the refrigerator. This sample group was coded as GBTF.

## 3. Experimental Methods

### 3.1. Determination of Active Components in Highland Barley Enzyme-Treated Beverage

#### 3.1.1. Determination of GABA

The extraction of GABA was conducted following the method outlined by Jannoey et al. with some modifications [6]. Anhydrous ethanol was used as the extraction solvent, and the sample was shaken in a 1:3 ratio of sample to extraction solution for 10 min. The mixture was then centrifuged at 4000 rpm for 5 min, and the supernatant was collected for further analysis. Next, 1 mL of sample/standard was mixed with 0.2 mL of sodium bicarbonate solution (0.04 g/mL) and 0.4 mL of 4-dimethylaminoazobenzene-4′-sulfonyl chloride solution (2 mg/mL). The mixture was stirred and subjected to derivatization at 70 °C for 20 min. After cooling, it was filtered through a 0.2 μm organic membrane for further use. The HPLC detection conditions were set as follows: Mobile phase A consisted of a sodium acetate solution prepared by dissolving 3.689 g of sodium acetate in 900 mL of water, which was then filtered. Mobile phase B was chromatographic acetonitrile. An Agilent 20RBAX SB-C18 column (4.6 × 250 mm, 5 μm) was used. The flow rate was maintained at 1.0 mL/min. The detection wavelength was set at 436 nm, the column temperature was 30 °C, and the injection volume was 20 μL. The elution program was as follows: 0–30 min, 73% A, 27% B; 30.01–40 min, 30% A, 70% B; 40.01–45 min, 73% A, 27% B.

#### 3.1.2. Determination of Free Amino Acid

We utilized the Solarbio Amino Acid Assay Kit (BC1575) to determine the amino acid content [15]. We modified the sample extraction process to aspirate 0.1 mL of the sample and 0.5 mL of reagent 1. We followed the instructions provided in the kit manual for other procedures and result calculations. No sample dilution was performed.

#### 3.1.3. Determination of Total Phenols and Flavonoids

We employed the Nanjing Jiancheng Bioengineering Research Institute Total Phenol Assay Kit (A143-1-1) and the Solarbio Flavonoid Content Assay Kit (BC1335) to measure the levels of total phenols and flavonoids in the samples, respectively. We conducted the experiments and performed calculations in accordance with the instructions provided in the respective assay kit manuals. Briefly, 0.1 g of barley drink was weighed; 2 mL of 60% ethanol solution was added, crushed for 5 s, reduced from 8 s, extracted by ultrasonic extraction for 30 min (300 W, 60 °C), and centrifuged at 4000 rpm and 25 °C for 10 min; and the supernatant was taken to measure the total phenol content. Slightly differing from the method of Wang et al. [16], 0.1 g of barley drink was weighed, 1 mL of 60% ethanol solution was added, ultrasonic extraction was carried out for 30 min (300 W, 60 °C) at 12,000 rpm and 25 °C, centrifugation was performed for 10 min, and the supernatant was fixed to 1 mL with 60% ethanol solution to be measured. The standard curve was plotted with rutin standard to calculate the flavonoid content, and the obtained standard curve was y = 0.7288x − 0.0031, with R^2^ = 0.998.

### 3.2. Determination of Antioxidant Capacity in Highland Barley Enzyme-Treated Beverage

We utilized colorimetry kits from the Nanjing Jiancheng Bioengineering Research Institute (A015-1, A052-1-1, A018-1-1) to individually measure the total antioxidant capacity, superoxide anion clearance ability, and hydroxyl free-radical clearance ability of the samples. We conducted the experiments and performed calculations in accordance with the instructions provided in the respective assay kit manuals [17,18]. We employed the Solarbio assay kit (BC4750) to assess the DPPH free-radical clearance capacity of the samples. The sample extraction process was modified to include the aspiration of 0.6 mL of the sample and 0.4 mL of the extraction solution. The mixture was vortexed and centrifuged at 10,000 rpm for 10 min at room temperature, and the supernatant was collected and placed on ice for analysis. Vitamin C was used as a positive control. We followed the instructions provided in the kit manual for the other procedures and result calculations. No sample dilution was performed.

### 3.3. Sensory Quality Evaluation of Barley Enzyme-Treated Beverage

#### 3.3.1. Determination of Taste and Odor by Electronic Tongue and Nose

Previous studies have detailed the TS-5000Z electronic tongue (Insent Inc., Atsugi-Shi, Japan) and the PEN3.5 electronic nose (Insent Inc., Kyoto, Japan), with slight modifications to the measurement methods [19]. When utilizing the electronic tongue, the sample injection volume amounted to 35 mL, the data collection duration was set at 120 s with a sampling frequency of 1 s, and the cleaning duration lasted 10 s. When employing the electronic nose, 10 mL of the sample was accurately measured, 3 g of sodium chloride was added, the mixture was sealed in a 50 mL bottle, and it was immersed in a water bath at 50 °C for 30 min before testing. The sample preparation took 5 s; sensor cleaning persisted for 120 s; sensor zeroing required 10 s; the injection rate was 400 mL/min; analysis sampling took 90 s; and feature extraction spanned 57–59 s.

#### 3.3.2. Determination of Volatile Components

We combined 5 mL of the sample and 3 g of NaCl within a 20 mL headspace vial, followed by subjecting the mixture to a 5 min thermal shock at 50 °C. The adsorption–desorption process and quantification method were consistent with a previous study [19], but the temperature profile of the GC was modified as follows: it was initiated at 40 °C and held steady for 2 min, increased to 90 °C at a rate of 2 °C/min, and further raised to 250 °C at 10 °C/min, where it was kept for 5 min. The ion source temperature was set at 230 °C, and the quadrupole temperature was set at 250 °C.

### 3.4. Statistical Analysis

All data were subjected to a one-way analysis of variance (ANOVA) using IBM SPSS Statistical 26. Statistical significance was defined as *p* < 0.05. Data analysis and plotting were performed using Excel, GraphPad Prism (version 8.3.0), and Origin 2021.

## 4. Results and Discussion

### 4.1. The Impact of Different Treatments on the Active Components in Highland Barley Enzyme-Treated Beverages

#### 4.1.1. GABA and Amino Acid Content

**Figure 1:**
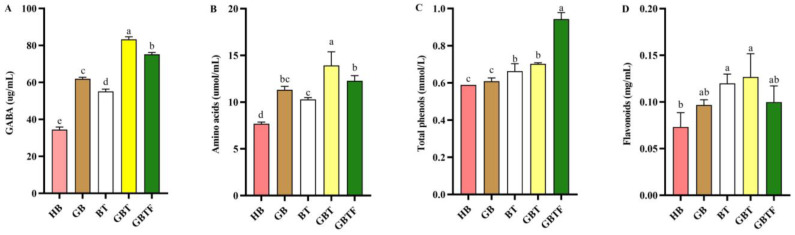
The impact of different treatments on the (**A**) GABA, (**B**) amino acids, (**C**) total phenols, and (**D**) flavonoid content in highland barley enzyme-treated beverages. Highland barley (HB), germinated barley (GB), barley with TB13 (BT), germinated barley with TB13 (GBT), and germinated barley with TB13 added and then fermented (GBTF). The results are the mean of three samples ± standard deviation. Different letters in the same column (a–e) represent significant differences (*p* < 0.05).

GABA is a valuable four-carbon free amino acid that has many health and nutrition benefits [20]. As shown in Figure 1A,B, compared to the HB sample, the GABA and amino acid content significantly increased in all the pretreated samples. The GABA content ranged from 34.46 to 83.28 ug/mL, and the amino acid content ranged from 7.68 to 12.29 umol/mL, with a consistent increasing trend. Germination, enzymatic hydrolysis, and fermentation all facilitate the breakdown of proteins into amino acids under the action of proteases, and these amino acids can promote GABA synthesis through multiple amino pathways [10]. Among them, the GBT sample, treated by germination and the addition of TB13 barley powder during the enzymatic hydrolysis process, exhibited the highest GABA and amino acid content. Germination treatment led to an 80% and 47% increase in GABA and amino acid content, respectively. It has been reported that germination can cause glutamate decarboxylase to convert glutamate to GABA [21]. The addition of TB13 resulted in a 60% and 34% increase in GABA and amino acid content, respectively, clearly demonstrating that highland barley with elevated GAD activity facilitates GABA enrichment [11]. However, the effect of germination treatment was significantly superior to the addition of active barley powder. Compared with the GBT sample, the GBTF sample, after fermentation treatment, showed a significant decrease in GABA and amino acid content, but both were still significantly higher than in the GB and BT samples. Although lactic acid bacteria can synthesize GABA during fermentation [22], the amino acid molecules obtained through germination and enzymatic hydrolysis may also be directly utilized by the bacteria, resulting in the lower GABA content in the GBTF sample compared to the GBT.

#### 4.1.2. Total Phenol and Flavonoid Content

Phenolic compounds are important metabolites in barley, typically including free phenols and bound phenols. Phenols bound to cellulose in the seed coat are crucial defense mechanisms for seeds; hence, the content of bound phenols is usually higher than that of free phenols [23]. Bound polyphenols mainly involve chemical bonds such as ester bonds, glycosidic bonds, ether bonds, etc., with large molecules like proteins, cellulose, and plant matrices. They are physically trapped in the plant matrix or embedded in cell structures [24]. Different treatments such as germination, enzymatic hydrolysis, microwave-assisted extraction, and fermentation can disrupt the structure of bound phenols, thereby altering the content and composition of phenolic compounds in barley [25,26]. For example, lignin in seeds can be degraded into hydroxybenzoic acid and vanillin under enzymatic reactions, increasing the content of free phenols [27].

As shown in Figure 1C,D, the total phenol content increased in all the pretreatments, except for the GB sample, where the other treatments significantly elevated the total phenol content of the beverages. The total phenolic content increases gradually with the duration of germination, and it is possible that a shorter duration of germination leads to a smaller increase in phenolics [28]. Among the samples, the total phenol content in the GBTF sample increased the most, from 0.59 mmol/L in HB to 0.94 mmol/L, representing a 60% increase. This result is consistent with the findings of Al-Ansi [29] and others, who observed an increase in total phenol content through germination and subsequent fermentation. Under the influence of fermentation, the regular degradation of cell wall-degrading enzymes, such as esterases, on these linkages leads to the release of new phenolic compounds during the pretreatment process [30]. Meanwhile, the total phenol content in the BT sample was higher than that in the GB sample, indicating that the added TB13 released more phenolic substances during the enzymatic hydrolysis process. However, the total phenol content in the GB sample also slightly increased, possibly because essential enzymes in phenolic biosynthesis, such as phenylalanine ammonia-lyase, could be activated during germination [31]. As shown in Figure 1D, compared to the HB sample, the flavonoid content in the GB, BT, GBT, and GBTF samples increased by 37%, 62%, 76%, and 40%, respectively. However, notably, the flavonoid content in the fermented GBTF sample significantly decreased compared to the nonfermented GBT sample. Some studies suggest that fermentation by *Lactobacillus plantarum* can metabolize complex flavonoids into simpler compounds [32]. In summary, through four different processing methods, the content of total phenols and flavonoids in barley enzyme-treated beverages can be increased.

### 4.2. Impact of Pretreatment on Highland Barley Beverage Antioxidants

**Figure 2:**
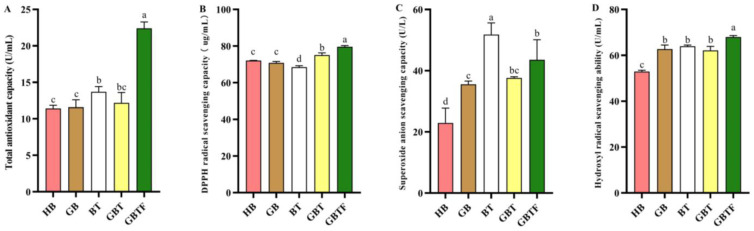
The effects of various treatments on (**A**) total antioxidant capacity, (**B**) total antioxidant capacity, (**C**) superoxide anion scavenging capacity, and (**D**) hydroxyl radical scavenging ability in enzyme-treated highland barley beverages. Highland barley (HB), germinated barley (GB), barley with TB13 (BT), germinated barley with TB13 (GBT), and germinated barley with TB13 added then fermented (GBTF). The results are the mean of three samples ± standard deviation. Different letters in the same column (a–d) represent significant differences (*p* < 0.05).

As shown in Figure 2, compared to the HB sample, the GB sample had significantly increased ability to eliminate superoxide anions and hydroxyl radicals. Germination treatment aids in the release of phenolic compounds, thereby enhancing the antioxidant activity of highland barley [12]. It was reported that ferulic acid contributed the most to ABTS activities [33]. Meanwhile, the findings of Fogarasi et al. [34] indicate that no significant increases in DPPH and total antioxidant capacity were observed during wheat malting, which is consistent with the results of this study. In the case of the BT sample, apart from a significant decrease in DPPH free-radical scavenging capacity, the other three antioxidant activities were significantly enhanced, with a remarkable 126% increase in superoxide anion scavenging capacity. The GBT sample showed a significant improvement in its DPPH free-radical, superoxide anion, and hydroxyl radical scavenging capacities. Meanwhile, the GBTF sample exhibited a significant increase in all four antioxidant capabilities, with a notable 96% enhancement in total antioxidant capacity. There is a significant positive correlation between the total phenol content and total antioxidant capacity of barley enzyme-treated beverages. Phenolic compounds with higher antioxidant activity in barley include Prodelphinidin B3, catechin, epicatechin gallate, and luteolin-3-ol [35]. Phenolic compounds in barley itself can directly participate in scavenging free radicals, transforming peroxyl radicals into stable byproducts, or indirectly clearing free radicals by inhibiting enzymes that can generate free radicals [13]. While the GBTF sample ranked second in terms of superoxide anion scavenging capacity, lower than the BT sample, it took the top position in terms of the other three antioxidant activities, indicating that fermentation significantly enhanced the antioxidant activity of barley enzyme-treated beverages.

### 4.3. Effect of Pretreatment on Sensory Quality of Highland Barley Beverages

Sensory evaluation has been accomplished through visual observation, the use of taste buds in the mouth, and the olfactory sense of the nose. However, physiological differences and variations in olfactory abilities among individuals can lead to inaccurate and non-reproducible results. Furthermore, extended periods of tasting can result in sensory fatigue or desensitization of the human sensory organs. The electronic tongue and electronic nose boast several merits, including robust repeatability, swift measurements, and user-friendly operation [36].

**Figure 3:**
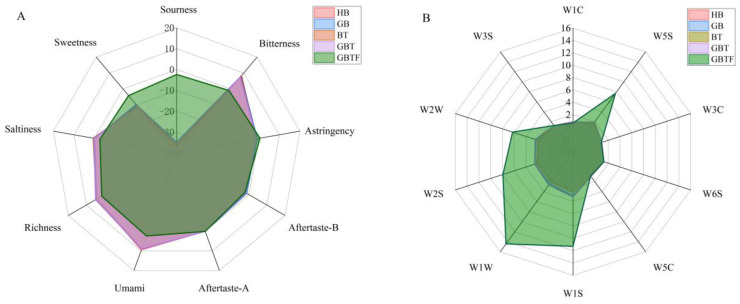

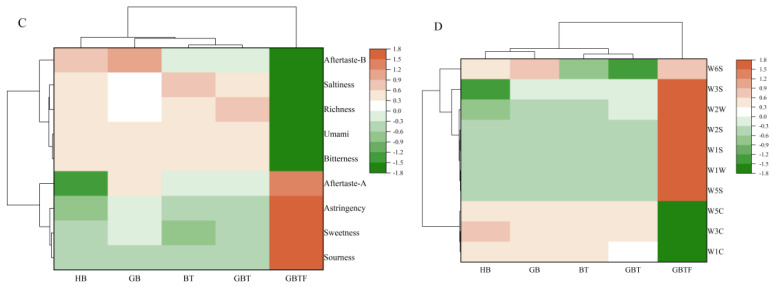
The radar chart (**A**) and cluster heatmap (**C**) for barley enzyme-treated beverages treated with different methods using an electronic tongue. Additionally, it presents the radar chart (**B**) and cluster heatmap (**D**) for the same samples analyzed with an electronic nose. The results are the mean of three samples ± standard deviation. Highland barley (HB), germinated barley (GB), barley with TB13 (BT), germinated barley with TB13 (GBT), and germinated barley with TB13 added then fermented (GBTF).

The taste and aroma profiles of the HB, GB, BT, and GBT samples overlap, with similar taste characteristics and aromas, as depicted in Figure 3A,B. The HB, GB, BT, and GBT samples exhibit higher levels of freshness, saltiness, and richness compared to the GBTF samples, which notably lack acidity. In contrast, the enzymatically treated barley beverage GBTF, obtained through fermentation, shows an increase in acidity and a decrease in bitterness. It is possible that *Lactobacillus plantarum* utilized certain bitter amino acids to produce a variety of organic acids [37]. The olfactory profile of the GBTF samples significantly expanded, with sensory sensors W1S, W2S, and W5S exhibiting a marked increase in response. Similarly, sensors W1W and W2W also showed a significant rise in response. GBTF was metabolized by microorganisms to alter and produce more aromatic compounds, volatile sulfides, and nitrogen oxides [38]. This indicates that fermentation not only effectively changes the mouthfeel of barley enzyme-treated beverages but also generates more volatile compounds, thereby improving the overall flavor of the barley enzyme-treated beverages.

As illustrated in Figure 3C,D, taste sensors classify acidity, sweetness, astringency, and astringency aftertastes into a single category. The HB, GB, BT, and GBT samples exhibit similar and generally lower response values compared to the GBTF samples. Conversely, bitterness, freshness, richness, saltiness, and bitterness aftertastes are grouped together, with the HB, GB, BT, and GBT samples showing similar and generally higher response values than the GBTF samples. Olfactory sensors cluster W5C, W3C, and W1C, all responsive to aromatic compounds, into one category. The HB, GB, BT, and GBT samples show similar and overall higher response values compared to the GBTF samples. The W5S, W1W, W1S, W2S, W2W, W3S, and W6S sensors are grouped together, indicating that the HB, GB, BT, and GBT samples have similar and overall lower response values compared to the GBTF samples. Horizontal clustering reveals that, whether in taste or aroma features, the HB, GB, BT, and GBT samples are more closely grouped, while the GBTF samples exhibit clear distinctions from other samples. Further analysis reveals that germination significantly altered the taste of HB and GB beverages without the addition of TB13. In contrast, for the BT and GBT samples with TB13, germination had little impact on taste. Notably, the effects of germination and the addition of active barley powder on the taste and aroma characteristics of the beverages vary. Germination had a minor influence on aroma, whereas the addition of TB13 caused substantial changes, leading to the grouping of HB and GB into one cluster and BT and GBT into another. The addition of TB13 likely contributed significantly to the taste and aroma, making it well expressed in the beverages.

### 4.4. Impact of Pretreatment on Volatile Flavor Compounds in Highland Barley Beverages

**Figure 4:**
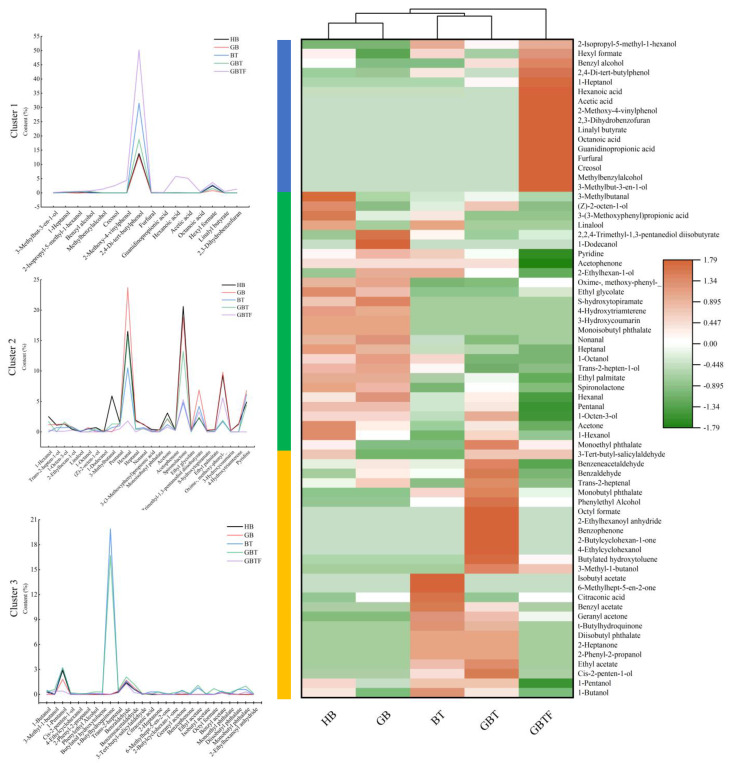
The cluster heatmap of volatile compounds in barley enzyme-treated beverages treated with different methods. Highland barley (HB), germinated barley (GB), barley with TB13 (BT), germinated barley with TB13 (GBT), and germinated barley with TB13 added then fermented (GBTF).

**Figure 5:**
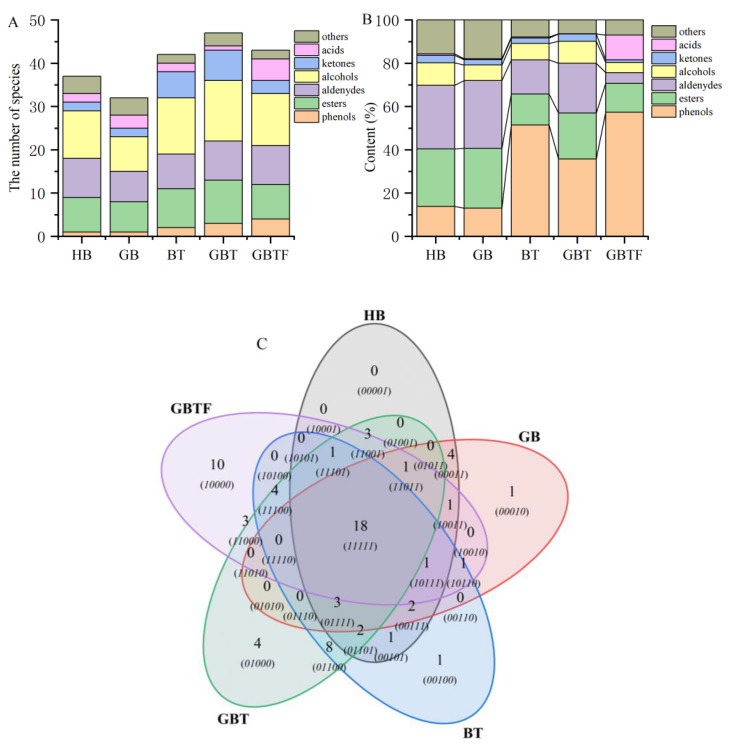
The types (**A**), concentrations (**B**), and a Venn diagram (**C**) of volatile components in barley enzyme-treated beverages treated with different methods. Highland barley (HB), germinated barley (GB), barley with TB13 (BT), germinated barley with TB13 (GBT), and germinated barley with TB13 added then fermented (GBTF).

As shown in Figure 4, GC-MS analysis successfully identified 69 compounds in the five sample groups, including 14 esters, 20 alcohols, 7 acids, 10 aldehydes, 7 ketones, 5 phenols, and 6 other compounds. The vertical clustering in Figure 4 aligns with the results of the electronic nose and electronic tongue analyses, highlighting the significant differentiation of GBTF samples in terms of their volatile flavor compounds compared to the other four groups. Combining the analysis of substance composition in Figure 5A,C, the five sample groups contain 37, 32, 42, 47, and 43 volatile compounds, respectively, with alcohols, esters, and aldehydes being the predominant categories.

The common compounds across all five sample groups include acetone, 1-pentanol, 1-octen-3-ol, 1-heptanol, 2-ethylhexanol, 2,4-di-tert-butylphenol, pentanal, hexanal, heptenal, nonanal, benzaldehyde, benzeneacetaldehyde, acetophenone, oxime-methoxy-phenyl-, hexyl formate, spironolactone, benzyl acetate, and 2,2,4-trimethyl-1,3-pentanediol diisobutyrate. Alcohols such as 1-pentanol, 1-heptanol, and 2-ethylhexanol contribute to the pleasant, mellow, and fruity flavor characteristics of barley grains. 1-octen-3-ol imparts a mushroom-like aroma, while aldehydes like pentanal, hexanal, heptenal, nonanal, benzaldehyde, and benzeneacetaldehyde provide malt, grass, fatty, and rose aromas [39].

The GB, BT, and GBT samples obtained one, one, and four additional compounds, respectively, through germination and the addition of active barley powder. These include 1-dodecanol, isobutyl acetate, 4-ethylcyclohexanol, octyl formate, 2-butylcyclohexan-1-one, and 2-ethylhexanoyl 2-ethylhexanoate. However, the GBTF samples exhibited an increase in 10 new substances, with acids being the most prevalent category. These acids include acetic acid, octanoic acid, and guanidinopropionic acid, contributing acidity and forming esters through reactions with ethanol, enhancing the overall flavor of the beverage [40]. The addition of furfural, originating from the thermal decomposition of polypentose in cereal husks, provides an almond-like aroma and sweetness, significantly enhancing the rich caramel flavor of barley enzyme-treated beverages [41]. 2-Methoxy-4-vinylphenol imparts a vanilla flavor to the barley enzyme-treated beverages. Enzymatic hydrolysis, fermentation, and other treatments break down proteins in barley into amino acids, which are crucial precursor substances for the formation of flavor compounds [38].

As shown in Figure 4, the vertical clustering of barley enzymatic beverages treated in different ways reveals consistent results when comparing volatile flavor compounds measured by GC-MS and aroma characteristics assessed by the electronic nose during sample clustering. The GBTF sample was distinguished from the others, while the HB and GB samples and the BT and GBT samples were further separated. This clearly demonstrates that fermentation significantly altered the flavor of the barley enzymatic beverages, while the addition of TB13 active barley powder also contributed to flavor changes, though its effect was weaker than that of fermentation. Germination, on the other hand, had minimal impact on the flavor of the beverages. From the horizontal clustering, the volatile flavor compounds in the beverages were mainly grouped into three categories. The first category contained 16 compounds, with their concentrations being higher in the GBTF sample compared to the others. Notably, 2,4-di-tert-butylphenol and hexyl formate exhibited significant differences in concentration due to varying treatments, such as germination, TB13 addition, and fermentation. Hexanoic acid and acetic acid were fermentation products, and in the fermented GBTF sample, the relative content of acetic acid and hexanoic acid reached 5.1% and 5.8%, respectively. The second category comprised 26 compounds, characterized by significant variability in concentration depending on the treatment method. The third category included 27 compounds, where 1-pentanol, t-butylhydroquinone, benzaldehyde, ethyl acetate, octyl formate, diisobutyl phthalate, and monobutyl phthalate were found in higher concentrations in the BT or GBT samples, which was potentially related to the addition of highly active TB13 barley powder.

As shown in Figure 5A,B, the number of volatile compounds in the different samples ranged from 32 to 47, consisting of esters, aldehydes, phenols, and other similar substances, with a relatively similar composition structure. In contrast, the total content of volatile flavor compounds such as phenols and esters in the germinated GB sample was close to that in HB. However, in the BT, GBT, and GBTF samples with added active barley powder, there were significant changes in the composition of volatile flavor compounds, including an increase in phenols and a decrease in esters, aldehydes, and other substances. Fermentation notably increased the content of acids while reducing the content of aldehydes and ketones.

## 5. Conclusions

This study, through germination, enzymatic hydrolysis, the addition of active highland barley powder, and fermentation treatments, not only increased the content of GABA, amino acids, and phenolic compounds in highland barley beverages but also enhanced their antioxidant capacity. Additionally, it improved the flavor of highland barley beverages. Among the samples used, the GBT sample stood out for having the highest content of GABA, free amino acids, and total flavonoids. In contrast, the fermented GBTF sample exhibited a significant increase in total phenolic content and overall antioxidant capacity, reaching 1.6 times and 2 times that of the control sample, HB, respectively. The GB, BT, and GBT samples had a minimal impact on the taste and aroma of the enzymatically hydrolyzed highland barley beverages. However, the levels of compounds such as 2,4-Di-tert-butylphenol, hexyl formate, hexanal, acetone, spironolactone, 2,2,4-trimethyl-1,3-pentanediol diisobutyrate, and pyridine varied significantly between the treatments. Notably, the fermented GBTF sample drastically altered the flavor profile of the enzymatically hydrolyzed highland barley beverage, reducing its bitterness and increasing its acidity. This was accompanied by a significant rise in compounds such as hexanoic acid, acetic acid, octanoic acid, 2,4-di-tert-butylphenol, hexyl formate, and 1-heptanol. The findings of this research will contribute to the development of innovative barley-based functional beverages that combine enhanced nutritional benefits with improved sensory qualities, ultimately offering a valuable addition to the functional food market.

## Data Availability

The original contributions presented in the study are included in the article, further inquiries can be directed to the corresponding author.
